# Barriers and facilitators in the implementation of bio-psychosocial care at the primary healthcare level in South Kivu, Democratic Republic of Congo

**DOI:** 10.4102/phcfm.v13i1.2608

**Published:** 2021-04-20

**Authors:** Christian E.N. Molima, Hermès Karemere, Ghislain Bisimwa, Samuel Makali, Pacifique Mwene-Batu, Espoir B. Malembaka, Jean Macq

**Affiliations:** 1 École Régionale de Santé Publique (ERSP), Faculté de Médecine, Université Catholique de Bukavu, Bukavu, The Democratic Republic of Congo; 2 Institute of Health and Society (IRSS), Ecole de Santé Publique, Université Catholique de Louvain, Brussels, Belgium; 3 Ecole de Santé Publique, Université Libre de Bruxelles, Brussels, Belgium

**Keywords:** change, bio-psychosocial, primary care, barriers, qualitative research, DRC

## Abstract

**Background:**

In the Democratic Republic of Congo (DRC), healthcare services are still focused on disease control and mortality reduction in specific groups. The need to broaden the scope from biomedical criteria to bio-psychosocial (BPS) dimensions has been increasingly recognized.

**Aim:**

The objective of this study was to identify the barriers and facilitators to providing healthcare at the health centre (HC) level to enable BPS care.

**Settings:**

This qualitative study was conducted in six HCs (two urban and four rural) in South-Kivu (eastern DRC) which were selected based on their accessibility and their level of primary healthcare organization.

**Methods:**

Seven focus group discussions (FGDs) involving 29 healthcare workers were organized. A data synthesis matrix was created based on the Rainbow Model framework. We identified themes related to plausible barriers and facilitators for BPS approach.

**Results:**

Our study reports barriers common to a majority of HCs: misunderstanding of BPS care by healthcare workers, home visits mainly used for disease control, solidarity initiatives not locally promoted, new resources and financial incentives expected, accountability summed up in specific indicators reporting. Availability of care teams and accessibility to patient information were reported as facilitators to change.

**Conclusion:**

This analysis highlighted major barriers that condition providers’ mindset and healthcare provision at the primary care level in South-Kivu. Accessibility to the information regarding BPS status of individuals within the community, leadership of HC authorities, dynamics of HC teams and local social support initiatives should be considered in order to develop an effective BPS approach in this region.

## Background

Person-centred healthcare is defined as individual care that considers needs, values and choices of people. A person-centred approach ensures, amongst others, that an individual is actively involved in therapeutic decisions.^1,2^ An important condition for person-centred care (PCC) is the communication and mutual trust between healthcare providers and people. Person-centred care could also cover the spectrum of bio-psychosocial (BPS) health.^3,4^

Enlarging the perspective of individual healthcare beyond diagnosis and treatment of disease also implies looking at one’s psychological, social, somatic and spiritual capacities.^5^ They are potential resources to help people to cope with environmental stress or diseases.^6^ In the BPS approach, interaction between individuals, within families and community groups is therefore important.^7^

Ensuring that this approach is followed implies that services provided by primary healthcare should then aim at strengthening community solidarity in addition to caring for individuals. Therefore, to achieve this, the operationalisation of primary healthcare needs to be framed in a different way, based on the broad BPS capacities and needs of individuals within a given community.^8^

Democratic Republic of Congo (DRC) has a longstanding history of progressive primary healthcare policies. It was one of the first French-speaking African countries to promote and implement the primary healthcare approach where health centres (HCs) (primary care services) constitute one of the fundamental components of a district health system.^9^

Few studies report evidence on the implementation of a BPS approach in DRC. Some authors note that the health system remains mostly focused on disease control and mortality reduction for targeted groups of the population (e.g. maternal and child mortality).^10,11^ Most financing and performance measurement strategies still target the traditional priorities of disease (like diabetes, malnutrition) and targeted ‘at-risk’ groups.^12^

Against this background, a broad research project for development has been financed by Académie de Recherche et d’Enseignement Supérieur (ARES) in Bideka, Burhale, Kabushwa, Lwiro, Lumu and Nyamuhinga HCs in South-Kivu (Eastern DRC). It had two main components: firstly, to measure the BPS health of the community over time and, secondly, to assess the feasibility of implementing a package of interventions in HCs to move towards the BPS approach of care. This article reports a sub-study of the second component. It aimed, through the perception of HCs workers, at identifying the barriers and facilitators of moving from the usual approach of primary care service provision focusing on disease control and targeted actions towards specific socio-demographic groups, to an approach focusing on broader BPS dimensions in people’s lives at the HC level.

## Methods

### General description of the six health centres

South Kivu, in the Eastern part of DRC, has 34 health zones, which are considered as the operational level of the health system. Each health zone is divided into health areas (15 on average) organised around a HC (primary healthcare level of the DRC health system). Six HCs (two urban, Lumu, Nyamuhinga and four rural, Bideka, Burhale, Kabushwa, Lwiro) from four health zones (Bagira, Miti-Murhesa, Katana and Walungu) were selected for this study (Table 1). This choice was based on their accessibility as well as their partnership with non-governmental organisations (Louvain Cooperation) that are also part of a broader project on which this study draws.

**TABLE 1:** Description of the study environment in 2017 (health areas and zones).

Health zone (HZ)	Population by HZ (2017)	Number of health area by HZ	Population by health area considered in study (2017)	Number of health worker by health centre	Utilisation rate per facility (number of cases received × 100/number of cases expected) (%)
Bagira	135.171 inhabitants	9	Lumu: 17.279 inhab.	13	16.4
			Nyamuhinga: 18.693 inhab.	13	33.7
Katana	222.951 inhabitants	18	Kabushwa: 19.225 inhab.	13	47.0
Miti-Murhesa	253.698 inhabitants	18	Lwiro: 10.900 inhab.	11	50.8
Walungu	268.434 inhabitants	23	Bideka: 10.340 inhab.	12	53.0
			Burhale: 19.295 inhab.	12	39.2

HZ, health zone.

### Organisational frame of analysis

**FIGURE 1:**
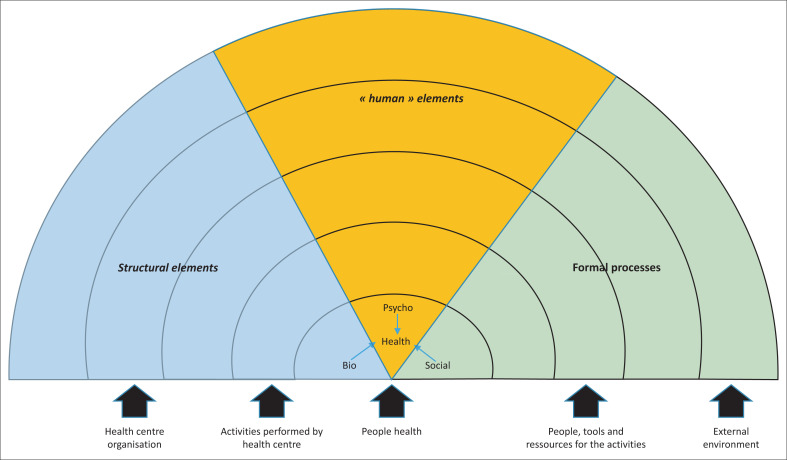
The Rainbow model adapted to bio-psychosocial considerations at the health centre.

We conducted a qualitative study in which we developed a framework to organise data collection inspired by the Rainbow model (Figure 1).^13^ Our framework describes different levels at stake in the HC development. The centre is occupied by people with their health described through its BPS dimension. The second level (clinical level) describes the activities performed at the HC such as patient consultation, home visit or community visit. The third level focusses on people (with specific skills), tools (clinical files) and resources (to perform home visits) that should be made available to strengthen the clinical level. The fourth level concerns HC organisation features such as quality development, regular staff meetings, complex cases discussion. Finally, the external environment should support changes at the HC level (i.e. through support from hospitals medical doctors…).

Different actions identified in this framework were used to organise data collection in HCs.

### Data collection

Data collection was conducted between November 2017 and February 2018. We organised seven focus group discussions (FGDs) with a total of 29 HCs staff to assess the challenges of the implemented BPS approach. The number of participants varied between five (Burhale, Lwiro, Nyamuhinga), six (Bideka, Lumu) and seven (Kabushwa) people per FGD; participants were selected by convenience. The choice of our sample was determined by the direct role that participants played in the care and follow-up of people who use the health services. The participants in the FGDs were nurses working in the HCs and nutritionists (some assuming the role of social workers) present in the health facilities at the time of data collection: their choice was motivated by the direct role they play in providing care at the HC and in the community. The FGDs were led by the principal investigator in a private room within the HC, after informed consent was obtained from each participant. The study objectives about BPS care implementation were explained to the different agents and the adapted Rainbow model (Figure 1) was used to structure the discussion (Appendix 1).

The principal investigator asked questions like ‘What do you mean by the BPS approach? What can make it possible to organize this approach at the HC or in the community?’. He also focused on possible challenges (according to different levels of Rainbow model) to improve the HC capacity to deal with complex BPS situations (i.e. that potentially combine somatic, psychological and social problems, such as people with multimorbidity and family problems or with stigmatisation problems) whilst an assistant took handwritten notes. All FGDs were conducted in French, recorded using an audio-recorder and transcribed into separate Microsoft Word files by an independent researcher for the project, which ensured the dependability of the data.

### Data analysis

Data extraction and synthesis were conducted using the levels of the adapted Rainbow Model. These included: (1) the meaning of ‘complex’ BPS situations; (2) the package of activities at the HC; (3) the organisation for the management of ‘complex’ BPS situations and community; the quality criteria and resources for services; (4) the HC management and (5) the influence of the environment (HC, community) on strengthening the management of HC. Verbatim statements were taken from each FGD transcript and were placed in the thematic groups by level. Each respondent was coded in a letter designating the HC and the number assigned according to the order of the interventions from the focus-groups. The 29 respondents were divided according to their response in A1 – A33 for Bideka, B1 – B30 for Lwiro, C1 – C36 for Nyamuhinga, D1 – D18 for Lumu, E1 – E35 for Burhale and F1 – F34 for Kabushwa. According to the order of verbatim for the analysis, the extracts from the focus-groups were chronologically numbered as A1 for the first intervention in Bideka, B20 for the 20th intervention in Lwiro.

After identifying, from the verbatim transcripts, the structural or human elements as well as the formal processes related to the working of HC team, two researchers located them at different levels of the rainbow frame. Then they identified forces at play that could influence changes towards the BPS form of care. Results were presented for validation of identified themes to the staff of all six HCs during a workshop to ensure the credibility of the data collected in healthcare facilities. This also contributes to increase trustworthiness and reflexivity.

### Ethical considerations

This research was approved by the Ethics Committee of the Catholic University of Bukavu (UCB) N UCB/CIE/NC/07/2017 and the Ethics Committee Hospital-Faculty Saint Luc-Catholic University of Louvain (UCL) n 2018/07FEV/046. An informed consent form was presented to each study participant and signed prior to data collection.

The Saint Luc-UCL Hospital-Faculty Ethics Committee has received and examined all of the documents relating to the aforementioned research project With CEHF reference: 2018/07FEV/046 and Belgian registration number: B 403.

## Results

Results are presented by levels related to the different parts of the Rainbow model discussed earlier. They are described as barriers found in most or all the HCs and specific facilitators found in some of the HCs. We illustrated each result with extracts from the statements of the people interviewed in the focus groups.

### People health

The perception that HC workers have of a BPS approach and the current way in which the same agents carry out community activities constitute barriers to the implementation of a BPS approach.

#### Understanding of a psycho-medico-social approach to health facility services provision by the centre

**The BPS approach was understood by health centre staff to be a new health program, similar to a mental health program:** Health centre staff considered that the BPS approach focused on mental health as a specific program and on the input (drugs and other resources) and training support required for it. They considered that the BPS approach had its place only in the health facilities controlled by the Ministry of Health (MoH) and they considered it to be a *new specific care program* in the same category as malnutrition or HIV programs.

In this case, the BPS approach is segmented into several components (medical, psychological and social) that do not have the same priority both for the health workers and the community: they are more interested in what is being supported, that is, with a focus on *mental health* at the expense of other components:

‘Yes, in my opinion I can say that it is a disease (a BPS situation) that requires assistance … after (medical) treatment one has to accompany him psychologically because he is traumatized; we must not leave it like this. We must also give him treatment psychologically. After treatment, we must also advise him.’ (B3, male nurse, 40 years)

### Clinical level

#### Activities performed by the health centre: Home visits in the community

Home visits were understood to be a means to sensitise the population and implement action for disease control.

When they are organised, home visits depend to a large extent on the dynamism of the community health worker since this role is devolved to them. Their actions in the community are more focused on awareness raising and disease control:

‘There are community health workers that we use a lot to look for these cases (malnutrition) …’ (C17, male nurse, 40 years)

‘When they (patients) come to the health center it is for the care and we also use the community health worker that will continue (providing care) in the community.’ (D17, male nurse, 44 years)

‘We succeed in this (home visits) via the community health workers … this is done with the community health workers in their health committee, …’ (E7, nurse, 48 years)

‘At the health center we can organize sensitizations, talk to them about their problems but what interests a lot are home visits.’ (E4, male nurse, 32 years)

#### Activities performed by the health centre: Creating forms of solidarity in the community

**The community is mentioned as a beneficiary of the services provided by the MoH and to which it has to conform … Endogenous forms of solidarity are barely mentioned, nor promoted:** For HC workers, community health workers (managed by MoH) are essential in raising community awareness, creating support (to MoH programs) and solidarity initiatives. The BPS approach is linked to them only if they follow directives imposed by the Health Ministry:

‘[… *W*]hen the project (mental health program) starts … the community health worker can know how they are in the community and how all those people suffer in villages …’ (A25, male nurse, 52 years)

‘We have community health workers who can help us …’ (B30, male nurse, 42 years)

Local health committees (at HC level), besides community health workers, are the other ‘master piece’ often mentioned.

The *local health committee* is more interested in activities carried out by the HC team, such as mass activities supported by donors with a financial incentive for community health workers.

Applying a BPS approach is only acceptable in health facilities integrated by the MoH: they are monitored and supervised by the hierarchy of the MoH and they must provide regular reports on their functioning and management of resources:

‘As they do not have this package (BPS approach), they will be obliged to transfer them (patients) to us …’ (B26, male nurse, 42 years)

‘It’s a specialty (mental health program) because we are in the same area but they (other healthcare facilities) do not have these activities that we do here.’ (C13, male nurse, 40 years)

Forms of solidarity are usually ‘created’ by external actors and focus heavily on diseases.

Mutual health insurance existing in health zones as well as patient associations that share a specific pathology are possible forms of solidarity, set by development international agencies or the MoH:

‘We have a committee of people living with HIV here, they get together, talk to each other, give advice and it works, … but for some committees we have not yet started a solidarity fund …’ (A22, male nurse, 50 years)

‘As our center is a pilot center, we started with senior citizens, if the partner (the donor agency) can also take other people, it can encourage the community …’ (C9, male nurse, 43 years)

‘There are those who have this disease (mental problem) but they are ashamed to get closer to the others of the health facility … If we could have the committee of these people who suffer from this problem (mental or psychological) and then among these people choose the committee that could sensitize, it could be better …’ (F29, nutritionist, 27 years)

### People, tools and resources

The constant use of additional training, additional external financial and human resources and limited access to people’s medical and social data constitute barriers to the implementation of a BPS approach in HCs.

#### Strengthening health centre services

**Health centre staff expect training, new resources and financial incentives:** According to HC staff, a new approach is not feasible without prior training of the different staff members, substantial financial and material resources:

‘We already have a little medical knowledge, but where we need help is what is required for psychological (treatment), and in the social area we would also like information about the activities income generating activities for example.’ (D4, male nurse, 44 years)

‘The continuous training of the staff would be really an asset and also inform other people …’ (A36, male nurse, 50 years)

‘As far as I am concerned, I think that it is necessary to be able, … For the medical aspect, we could follow (Ministry) norms but for the psychological aspects (we) need some capacitation …’ (F6, nurse, 43 years)

‘… Making inputs available, motivation … The bonus of the agents, the motivation can take different forms: there is the motivation through some training, there is the motivation through equipment.’ (D6, male nurse, 44 years)

The HC team must receive bonuses from donors to implement a new way of care even if its importance is evident for community members and their representatives:

‘It would also involve the need for donors for people that even a family member cannot help … if you can have a partner it will be a benefit to them.’ (B8, male nurse, 29 years)

‘[… *A*]nd we must also think about the motivation of this staff too because if you are well, if you are somehow well paid, we can do better also the work …’ (C21, male nurse, 43 years)

Health centre teams can only get involved when they receive financial support to organise these activities:

‘A motivation (financial) for example to the staff that consults cases, a small motivation of community health workers who are responsible for moving in the villages that would really accompany this activity, …’ (A9, male nurse, 50 years)

‘To get to visit someone who is 6 km away we face these difficulties in terms of means of transport … we try to do something and get help from the community health worker that also asks us something …’ (E14, male nurse, 32 years)

‘For us caregivers it will always ask the financial means to reach someone who lives a few kilometers …’ (E17, male nurse, 32 years)

Accountability (proof that training, resources and financial incentives are properly used) is understood as being ensured through health (zone) authorities’ supervision with specific indicators and format of reporting.

The role of the health zone’s core team is limited to supervision of new planned care activities in the health facilities:

‘… It is up to her (the health zone management team) to plan the background requests and she may also be receiving them and now we are executing and benefiting …’ (A26, male nurse, 50 years)

‘[… *T*]he zone can intervene by giving training via the partner and also with formative supervision …’ (B22, male nurse, 42 years)

A new approach to the health center involves additional support for reporting data and transmitting indicators to health authorities and donors:

‘We have a document in which we follow these cases … so we recorded it twice: we had the proper register of patients and a register that was used to accompany these cases …’ (F16, male nurse, 45 years)

‘[… *W*]e have consultation cards, there are many elements and in front of the patients we are eliminating them one by one … but the register is very important …’ (A12, male nurse, 50 years)

#### Structuring the patient’s medical file

**Agents describe very little or no use of a personalised medical record in health centres:** They reported that the support used for patient follow-up is not classified according to rules that allow details on the psychological and social components of the people who attend the HC:

‘[… *W*]e have a register that can be medical and another that can be psycho social, … two different registers …’ (A11, male nurse, 50 years)

‘[… *N*]o, they do not take into account psychological problems … not yet, and moreover, these are not forms, they are notebooks … I think that to follow these patients (with BPS problem) it would be necessary to be able to classify their files according to the source.’ (F16, male nurse, 45 years)

Registers are more common in use, with the primary function of reporting performances to authorities.

The trend is to use registers for the purpose of transmitting data and facilitating their reporting to hierarchy or health system donors:

‘[… *I*]n the register it’s just a small column for their identity and that’s all, … but the register does not contain all the elements that are found on the form …’ (A11, male nurse, 50 years)

‘You also need data collection tools, … registers; the consultation forms, the consultation sheets, the home monitoring sheets; because the person who is mentally affected must be followed at the level of the community.’ (C25, male nurse, 40 years)

The organisation of the permanence of the care teams in certain HCs and the archiving of the files of the people with detailed information on the people, their entourage constitute facilitators to the implementation of a BPS approach in HCs.

#### Availability of care teams

In some facilities (Bideka), usual working hours (from 7:30 PM to 3:30 PM as stipulated by the Congolese law) have been modified to allow health workers to receive patients earlier (between 6:30 AM and 7.30 AM) before they conduct other activities in the community.

The day service is extended to 4 PM to allow for follow-up of community members who have appointments with health workers:

‘[*M*]aybe not innovative but we have some aspects of competition, because here at the health center, we start working very early because we have a farming population … we took the option to start at 6.00 am, 6.30. The first to arrive, he is served, he returns to go to other activities, and the population tells us that it is really a good thing.’ (A38, male nurse, 50 years)

#### Accessibility to the individual information of the community

In two HCs (Nyamuhinga and Bideka), the medical information related to the patients who consulted is recorded in personalised notebooks kept at the HC.

These supports were classified according to a group that allowed each member of the community to be located according to the villages of origin, neighbourhoods and avenues. A long-term follow-up is possible by easily including the history of each patient:

‘Information on the patient, from his health center file, … that will help to know or to identify, which requires the home visit, in relation to their file … file correctly keeps all records of each patient, by avenue, by quarter, by street, by path, …’ (C26, male nurse, 43 years)

### Health centre organisation

The organisation of the sharing of experience in the teams of the HCs constitutes barriers to the implementation of a BPS approach in the HCs.

#### Case discussions at the health centre

The sparsely organised case discussions concern the medical problems of the patients observed at the health facility:

‘Regarding this discussion (cases) yes maybe we do it one way or another but we do not record it. Spontaneously we do our little staff meetings every Monday usually.’ (F18, male nurse, 45 years)

‘At service meetings we discuss cases and when the one who is equipped is with the patient, he calls others to see what he is doing.’ (B17, male nurse, 42 years)

Leadership of HC authorities can contribute as a facilitator to implementing the BPS approach.

#### Leadership of health centre authorities and dynamics of health centre teams

In some HCs (Bideka, Kabushwa), the leadership of the in-charge nurse is strengthened based on their seniority in that position (length of experience in that post), their ability to innovate, to make decisions made by the team and about the organisation of services and specific needs from the community. The stronger the leadership, the more the health facility develops in a certain way and the team is motivated to put in place local strategies for monitoring patients at the HC and in the community:

‘Those who can take care of these activities (BPS) are the nurses and at the head of the health centre is the in-charge nurse (titular nurse). At the head, the Titular Nurse organizes with the nurses and sees what they can do.’ (D3, male nurse, 44 years)

When decision-making is made collectively by the HC team in the organisation of services, the collaboration between the different agents is facilitated as along with the sharing of information on training in new modes of care:

‘So far, it’s collegial, it’s true in any organization there is often a head of department … but it’s really collegial it’s not to impose it, he or she (the titular nurse) may have a proposal that he or she can arrange to pass perhaps with friends … they are explained the merits of this proposal. If others accept it is admitted, if others find that it is not good we cancel, … at least that is how we work.’ (A15, male nurse, 50 years)

‘[*I*]f there was a training session and 2 nurses from the health center were invited for example, when they come from the training they have to brief the others, give them back so that they too are updated.’ (B9, nurse, 27 years)

### External environment

The interaction with the supervising doctors of the care structures constitutes barriers to the implementation of a BPS approach in the HCs.

#### Role of the referral physician at the health centre

**Health centre workers do not understand the role that the referring doctor should play:** The importance of this role and its relevance are not unanimously accepted amongst the staff of the HCs. There is a perceived risk that the actions of the medical doctor at the HC may compete with the tasks of the local team and the objective of the regular supervision visits.

The HC staff expressed the need for a clear definition of the role of reference level physicians in supporting the BPS approach at the HC:

‘[… *H*]e (the doctor) is going to break us … he’s going to ask us why we did this or that …’ (B27, male nurse, 42 years)

‘[… *T*]he doctor spends every Monday and Thursday on consultation only for older persons and a few special cases.’ (C18, male nurse, 43 years)

Local support in communities contribute as facilitators to implementing the BPS approach.

#### Local social support initiatives and the link with the community

In some health areas (Lumu, Bideka, Kabushwa), a special form of association between members of the community is reported (Association Villageoise d’Épargne et de Crédit or village savings and loans association). It is based on the principle of savings by contributions from members of the association and granting credit to one or more of them. Similar to mutual health insurance, this form of solidarity can provide support to people in precarious BPS situations:

‘[*W*]e can ask him to join one of the AVEC that exist like that in the framework of idea they can help each other in the AVEC that exists in the health area; … he can borrow in these AVEC … because it is an amount that is repayable little by little …’ (F24, nurse, 43 years)

Community health workers, as representatives of the community, set priorities in the health system with the HC staff. This allows them to better orient awareness on relevant topics in the community:

‘We do this (sensitization) via community health workers … this is done with the community relays in their health committee, … what are the priority problems of society in relation to chronic diseases for example …’ (E5, male nurse, 32 years)

## Discussion

The results of this research in six HCs in South Kivu, eastern DRC shed light on important elements that can make it easier or not to change the way usual activities of healthcare are implemented.

### Challenge of barriers to change the approach of care at the health centre level

The analysis of the conditions, in which these health facilities offer the current approach to care, sheds light on different characteristics that could hamper changes. These are related to peoples’ mindset and the traditional management of an organisation. It showed the importance of: (1) the central MoH’s influence as well as the international agencies that support the health system in the DRC, (2) the mindset of the health workers, including their perception of the provision of care and (3) the role of the community in the organisation of healthcare and social support.

Whilst contextual, those characteristics are an expression of the health system functioning in DRC. They are of particular interest because this fact has been rarely reported.^14,15^

Firstly, HC workers equate any suggested new approach with a vertical (in silo) program of disease control, such as programs to control malnutrition or diabetes.

Indeed, the health system in the DRC is still strongly anchored in a disease-centred approach, given the resources allocated to combat the major global scourges such as diabetes, malnutrition and the prevalence of infectious diseases.^16,17,18^

Secondly, broadening the scope on BPS dimensions as part of a person-centred approach in HCs is perceived, in the six HCs, as a series of additional activities that health workers must provide and for which (financial) incentives should be expected. The financial incentive factor has been noted in other studies as one of the factors limiting the involvement of health workers in change dynamics and their performance.^19^ It is essential to consider it in the process of change desired in the HCs. One of the strategies proposed would be to promote social mutuality between the health facility agents.^20^

Interviewees reported that proposing a new approach to patient care would necessarily require external support to the health system from non-governmental organisations (NGOs) and international donors. Providers from HCs are indeed used to execute activities decided and financed by outsiders, similar to what is observed in other parts of Sub-Saharan Africa.^21^

The resources from donors are given to managers of health zones who, in turn, finance certain activities planned in HCs. In this context, a HC without autonomous resources is not able to easily initiate and pilot a contextual change approach, inspired by the experience of the staff.^22^

Thirdly, interviewees reported that another factor that could make a change in the practice of care difficult in DRC would be the highly hierarchical decision-making process. This was observed in South Africa^19^ where the plans for care packages are not usually based on specificities of contexts of health areas. It is mostly the result of centralised decisions channelled, from district (the health zones in DRC), management level to peripheral level (HCs).

This mode of planning or management results in: (1) local stakeholders poorly empowered in the organisation of healthcare; (2) control procedures of HC supervisions (standardised and target-focused supervision) oriented by the MoH hierarchy and the donors.^19,23^ This makes it difficult for doctors from the referral hospital to play a real supportive role towards nurses (who perform most of the HC activities). There is evidence that HC workers can view the medical doctor more as a contender than a helper.

The doctor may be called to assume roles such as coaching local teams and providing support in the management of complex cases.^24,25^

Supervision by the reference level of primary health-care care agents should encourage collaboration between members of the same team to facilitate the transfer of knowledge. The desired change to a new approach could also involve the initial training of health workers on BPS considerations in the care approach.^3,26^

Fourth, home visits are usually organised in the community to raise awareness of prevention and health promotion aspects to combat certain diseases by targeting specific categories of the population. It was therefore difficult for HC staff to understand a possible role of psycho-social workers in primary care. This is worsened by the fear of HCs’ workers to see that their individual income decreases because of additional staff.

Community health workers in health areas could become a key link in the BPS approach, playing more of a role of community representatives. Their role would then be that of ‘experience experts’, better able to instil a psychosocial dimension, in addition to the biomedical dimension into the health care. They could then become agents of change in the relationship between professionals at the HC and in the community.^27,28,29^

### Bio-psychosocial care approach facilitators as grassroots initiative: What are possible solutions to a difficult change?

In our study, some HCs presented positively deviant characteristics like availability of human resources, local social networks or individual information. They could constitute an inspiration for curving HC changes towards a new form of BPS care. Indeed, they featured the possibility of better reorganising HCs and therefore, adapting care to the patient’s situation.

Some of the HCs are able to organise themselves so as to increase permanence of the care in the HC. As Draper and Engl mentioned, the availability of the health team and especially the attitudes displayed during care activities are essential factors that would likely create the atmosphere conducive to a BPS approach.^30,31^

At the same time, good HC organisation is often associated with strong leadership. Indeed, the leadership of some HC management teams was made visible through original initiatives mentioned by the participants in this study. Some of them targeted the organisation of individual and community information.

Team organisation and positive leadership were also reported by Moore et al. who acted as facilitators in implementing PCC.^32^ The organisation of the team is characterised by a distribution of tasks within the units of the health facility, participation in decision-making and transmission of information.^20^ By positive leadership we mean, altogether, expectations, objectives and values that make the management of a leader effective.^33^

Indeed, the management of the personal data of community members, as organised in some HCs, is essential in this context since it allows to maintain the continuity of care and to adapt the care according to the available BPS information.^34,35^

Finally, the social support provided through village savings and loans association (AVEC in French) in some villages was a good initiative to strengthen solidarity in communities. In other contexts, it would have facilitated and strengthened home support for people with complex BPS situations.^36,37^

### Limitations and strengths

Perceptions of community members and actors from the MoH and NGOs were not considered in this study as we focused on actors working in health facilities. Their views would be interesting to consider as they could help gain a better insight into their influence on the current functioning of the HC. Some of the study investigators came from outside the study areas, which might have influenced some of the comments made by study participants.

This work tried to explore the current functioning of the health system in the DRC, starting from the dynamism of the first line of care. From the perspective of the main actors who are the health workers, our findings highlight the current status of thought that conditions the various health system stakeholders and potential changes in healthcare services. Our study also shows how adapting the current care approach to a more personalised design may seem difficult.

### Implications

In the organisation process of the healthcare system in the DRC, the healthcare workers have been trained to effectively treat signs of diseases presented by patients in consultation without being interested in their full history, particularly in their social and psychological context, hence the need for alternative services to meet the expectations of community members.

Our study tried to understand how PCC by making use of a BPS approach can be organised at primary healthcare facilities and can help to identify characteristics that may limit or facilitate a change in healthcare provision habits. It shows that the organisation of care in South Kivu is very dependent on the contribution of donors outside the health system, and a very hierarchical relationship between the MoH and the agents involved in health activities. These elements must be considered in any change process to be proposed at the HC level.

## Conclusion

This study carried out in six HCs in South Kivu has illustrated possible fundamental rules that explain the current organisation of the primary healthcare level in DRC. It sheds light on prevailing forces that maintain stability in the HC despite expected changes. If provision of care has to change from a disease-centred to people-centred approach, implementation of a set of interventions is not enough. More attention needs to be paid to actors’ mindset, institutional arrangements and prevailing regimes, if any change is to occur. Although this appears difficult to achieve, gradual change can occur, especially if it is more often included in the logic of development projects and funding agencies.

## Appendix 1: Guide to interviewing the organisational analysis of health centers in the management of complex psychomedicosocial situations

Entretien approfondi avec partie prenante n

Ce guide d’entretien peut être adapté en fonction des personnes interviewées et de leur statut.

Essential points of research on the organization of the health care system in South Kivu and the management of psychomedicosocial situations

Je vous remercie de bien vouloir m’accorder quelques instants pour parler de l’actuelle organisation du système de soins dans la province du Sud Kivu en général et dans votre zone de santé plus précisément. Nous voudrions recueillir votre point de vue en ce sens.

Votre expérience par rapport au système de santé nous permettra de proposer une meilleure organisation de la prise en charge des patients avec multi morbidités. Vos idées et suggestions nous intéressent au plus haut point, n’hésitez pas à nous dire ce que vous en pensez vraiment.

Si vous le permettez, je souhaiterais vous parler d’une situation qui pourrait vous interpeller:

‘Il s’agit d’un paysan d’un village du Bushi, âgé de 45 ans, qui souffre de diabète depuis cinq ans. Ceci l’oblige à suivre un régime strict et à respecter un certain mode de vie. Lors d’un de ses examens de contrôle, l’infirmier lui signale que sa tension artérielle est élevée et qu’il doit pour cela changer de traitement et envisager des examens plus approfondis à l’hôpital général de référence. Les frais du traitement et des examens avoisinent les 100 dollars américains alors que le premier traitement lui revenait à 30 dollars. Il doit en même temps payer tous les frais scolaires pour son fils ainé qui passe l’Examen d’Etat un mois plus tard.’

Je m’en vais maintenant vous poser quelques questions:

1. Que pensez-vous de cette situation?
  1.1 Est-ce fréquent dans votre milieu? avec quel type de maladie?
  1.2 Comment pourriez-vous la décrire en quelques mots?
2. Certains peuvent décrire ce cas en parlant de situation psychomédicosociale complexe, qu’en pensez-vous?
3. Avez-vous déjà rencontré ce type de patients dans votre parcours professionnel? Comment les avez-vous pris en charge?
4. Pensez-vous que notre actuelle organisation des soins au niveau des centres de santé peut permettre de prendre en charge ce type de patients?
5. Qu’est ce qui peut être fait en plus selon vous au niveau du centre de santé?
6. Quel peut être le rôle de la communauté? de l’hôpital général? de la zone de santé? des partenaires?
7. Comment est-ce que votre proposition peut être intégrée de manière permanente dans les activités de votre centre de santé?
  - Qu’est ce qui peut garantir son succès?
  - Quels en sont les faiblesses?
  - Comment sera-t-elle financée?

Je vous remercie beaucoup pour le temps que vous m’avez accordé. Je reviendrai vers vous si les premières analyses exigeaient d’éclaircir certains sous points.
